# The Milk Supply in America

**Published:** 1922-11

**Authors:** 


### THE MILK SUPPLY IN AMERICA.

DR. ROBERTSON, the Medical Officer of Health for Birmingham, has issued a report to the Public Health Department of that city on the results of a visit undertaken by him for the purpose of seeing how the regulations in regard to the milk supply are carried out in the United States, and generally to ascertain on the spot what effect these regulations had in giving the people a good and wholesome milk supply at moderate price, and also whether they had any effect in increasing or otherwise the use of milk by the people. He finds that in America they are far in advance of this country in regard to the arrangements for the production of clean, wholesome milk and its distribution.

After pointing out that the only legal standard of quality of milk in England is that derived from the Food and Drugs Act, whereby it is presumed to be adulterated unless the contrary is proved when its fat-content is less than 3 per cent., he says that in most of the American cities the dairyman buys his milk to contain at least 3.5 per cent. of butter fat, with a proviso that for every 1/10th per cent. of butter fat above 3.5 per cent., extra will be paid at a rate which makes it worth while for the farmer to endeavour to get the largest amount of fat possible.

"There seems to me," says Dr. Robertson, "to be real grounds for asking the milk trade in Birmingham to purchase their milk from farmers on a fat basis somewhat analogous to that adopted in the States. Both the farmers and the public would greatly benefit if this were done. The farmers would get a better milk yield and a richer milk. But a change like this could not be made general at once —for the elimination of bad cows will be a slow process. The average yield per cow in England before the war was 550 to 575 gallons (5,637 to 5,894 lb. of milk). This is so low that it ought to be easily possible to increase it and at the same time to improve its composition."

Another quality basis made use of on a large scale in America is that of cleanliness, the cleanliness being estimated by the number of bacteria the milk contains. In some towns the distributor buys the milk on its bacterial content, very much on the same lines as he buys for fat content. As the number of bacteria in the milk falls below the maximum, a higher price is paid according to scale.

Dr. Robertson attaches great importance to the supply of milk in clean, sterilised glass bottles, and considers that the arrangements in Birmingham under which milk is at present bottled should be extended until all milk is so delivered. Another matter which he strongly commends is the American process of pasteurisation. It differs greatly from that used in Birmingham. In the first place, great care is taken that none of the milk comes in contact with a steam pipe or water pipe of high temperature. Care is taken that none of the milk is overheated while other portions are underheated. The overheated milk would be damaged, while the underheated portion would not be heated sufficiently to destroy bacterial life. In effect, they attempt to raise milk to a temperature of 145°F., without obtaining this degree of heat by mixing hotter and cooler milk together. Having carefully raised the temperature of milk to 145°F., they hold it at this temperature for half-an-hour in a large tank. At each stage the temperature of the milk is recorded by means of self-recording thermo-couples. The milk is then cooled by water and by means of a brine cooler to 40°F., at which temperature it is bottled and distributed.

"Everywhere we went," says Dr. Robertson, "in the towns of America it was possible to buy clean, rich, well-cooled milk in glass bottles at a price which is difficult to compare with that of milk in England but which undoubtedly was relatively lower. Milk which was dirty or of poor quality, or where the temperature of it was too high, was not allowed to be sold. The knowledge that clean, cold, rich milk could be obtained everywhere undoubtedly induced the people to drink more milk (the most valuable food we have)."

The whole of Dr. Robertson's report, to which this short review does scant justice, is a most interesting contribution to the vital question of improving the milk supply in this country and increasing the consumption of this article of food.

